# Adverse Effects of Sugammadex on the Cardiovascular System

**DOI:** 10.7759/cureus.34728

**Published:** 2023-02-07

**Authors:** Mamatha Kadiyala, Thomas Gedulig, Ratan K Banik

**Affiliations:** 1 Anesthesiology and Reanimation, University of Massachusetts Memorial Medical Center, Worchester, USA; 2 Anesthesiology, University of Connecticut, Bloomfield, USA; 3 Anesthesiology, University of Minnesota, Minneapolis, USA

**Keywords:** allergic reaction, allergic, cardiac arrest, allergy and anaphylaxis, sugammadex dosing and timing

## Abstract

Perioperative anaphylaxis is rare but potentially life-threatening. Although the most common causative agents are muscle relaxants and antibiotics, there have been several case reports of sugammadex-induced anaphylactic reactions. Though most cases of perioperative anaphylaxis present after induction, sugammadex anaphylaxis presents at the end of the case, sometimes in unmonitored situations such as after extubation or during transport to the recovery unit. Here we report a case of suspected sugammadex-induced anaphylaxis that led to cardiac arrest. We emphasize that vigilance is required when a high dose of sugammadex is used for the reversal of neuromuscular blockade.

## Introduction

In recent years, sugammadex has emerged as a common agent for the reversal of rocuronium- or vecuronium-induced neuromuscular blockade. The superiority of sugammadex over neostigmine has been shown in a meta-analysis of 13 randomized controlled trials with 1384 patients. Sugammadex was faster in reversing neuromuscular blockade, lowering the risk of postoperative residual paralysis, and was associated with a significantly lower likelihood of adverse effects and postoperative weakness [1]. Moreover, a recent Cochrane systematic review with a meta-analysis of 41 randomized controlled trials, including 4206 patients, confirmed that sugammadex caused 40% fewer adverse effects compared with neostigmine. Specifically, risks of bradycardia, postoperative nausea and vomiting, and overall signs of postoperative residual paralysis were reduced [2].

However, sugammadex has a propensity to cause anaphylaxis, which has been shown in multiple case reports and large retrospective data analyses [3]. A significantly higher incidence of sugammadex anaphylaxis compared with neostigmine is evident in the sixth National Audit Project study in the United Kingdom [4]. One confirmed case of sugammadex anaphylaxis is reported in an estimated 64,121 administrations [4], and none were reported in approximately 648,470 administrations of neostigmine. In another study, six cases of sugammadex-induced anaphylaxis were reported out of 29962 patients [3]. It is important to report suspected anaphylaxis cases to understand the incidence, clinical manifestation, diagnoses, and treatment. This knowledge may increase awareness and improve the safety of perioperative care. Here we report a case of suspected sugammadex-induced anaphylaxis that caused cardiac arrest. Written authorization from the patient was obtained to publish this case report.

## Case presentation

A 69-year-old female, ASA 3, with a past medical history significant for hyperlipidemia, hypertension, esophageal reflux, breast cancer, multinodular goiter, Langerhans cell histiocytosis, emphysema, pulmonary nodules, peripheral vascular disease, and coronary artery disease presented to Hartford Hospital (Hartford, Connecticut, USA) for transurethral resection of bladder tumor. She is an active smoker with a 40-pack-year history, exercises regularly, and is able to climb two flights of stairs (metabolic equivalent >4) without symptoms of chest pain. She was 5 feet 2 inches tall and weighed 82 kg and was allergic to amlodipine which leads to swelling. On physical examination, she was alert and appropriate, with a normal cardiac and pulmonary exam with a Mallampati score of two on airway exam. Her electrocardiogram showed normal sinus rhythm with no acute ST-T changes, and she was cleared for surgery by her cardiologist. All her labs were within normal limits. Her pre-procedure vitals were blood pressure 119/58, pulse 72, and SpO2 95%. Given her poorly controlled acid reflux, the decision was made to proceed with general anesthesia with a modified rapid sequence intubation. The patient was induced with 100 mcg of fentanyl, 50 mg of lidocaine, 200 mg of propofol, and 40 mg of rocuronium. A 7.0 mm endotracheal tube was placed using direct laryngoscopy with a Mac 3 blade. Two grams of cefazolin was given before the procedure started.

The procedure was completed 15 minutes earlier than anticipated. Neuromuscular monitoring showed no twitches; therefore, 320 mg of sugammadex (4 mg/kg) was administered. Four minutes later, while she (still intubated) developed profound hypotension with systolic blood pressure in the range of 40-50 mm of Hg. A diffuse erythematous rash in the trunk and an increase in peak airway pressures to 48-50 cm of water were noted. To stabilize blood pressure, an intermittent bolus of phenylephrine 100 mcg was administered without any effect (a total of 800 mcg was given). No carotid pulse was noted, and non-invasive blood pressure cuff cycling showed no reading. The patient also started desaturating to a SpO2 of 85%.

A code was called. Cardiopulmonary resuscitation was started with brief chest compressions, and 1 mg of epinephrine was given intravenously. With the return of pulsatile flow, an arterial line and large bore intravenous line were placed, and the patient was resuscitated with 2 liters of crystalloids. Arterial blood gas showed a pH of 7.28, pCo2 of 54 mm Hg, pO2 of 68 mm Hg with a base excess of -1. Intravenous diphenhydramine 50 mg, 100 mg hydrocortisone, and nebulized albuterol were also administered.

The patient needed repeat doses of vasopressin to treat resistant hypotension, and epinephrine infusion was started at 0.02 mcg/kg/min. Norepinephrine infusion was also started at 4 mcg/min, and the patient was weaned off this once she became hemodynamically stable on epinephrine. A serum tryptase level was sent to the lab within one hour of the development of hypotension. The patient was transported to the intensive care unit and maintained on propofol and epinephrine infusions. She was weaned off the epinephrine infusion within a few hours in the intensive care unit. However, she developed lactic acidosis due to the cardiac arrest.

The patient was extubated the following morning after confirmation of a cuff leak, and visual inspection showed an absence of glottic edema. She was discharged the following day from the intensive care unit in stable condition. Her serum tryptase level came back within normal limits (<11 mcg/L).

It was discovered later, upon further questioning, that the patient had a previous video-assisted thoracoscopic surgery two years ago for a lung biopsy in which she developed an erythematous rash in the post-anesthesia recovery unit. Her rash resolved after treatment with diphenhydramine. 

Her skin prick allergy test to cefazolin was negative. Allergy testing for sugammadex was not done. Given the temporal relation of her symptoms, sugammadex was labeled as an allergy, and the patient was counseled to alert anesthesiologists and perioperative team in future encounters. Since her discharge, the patient has been doing well and has quit smoking.

## Discussion

**Figure 1:**
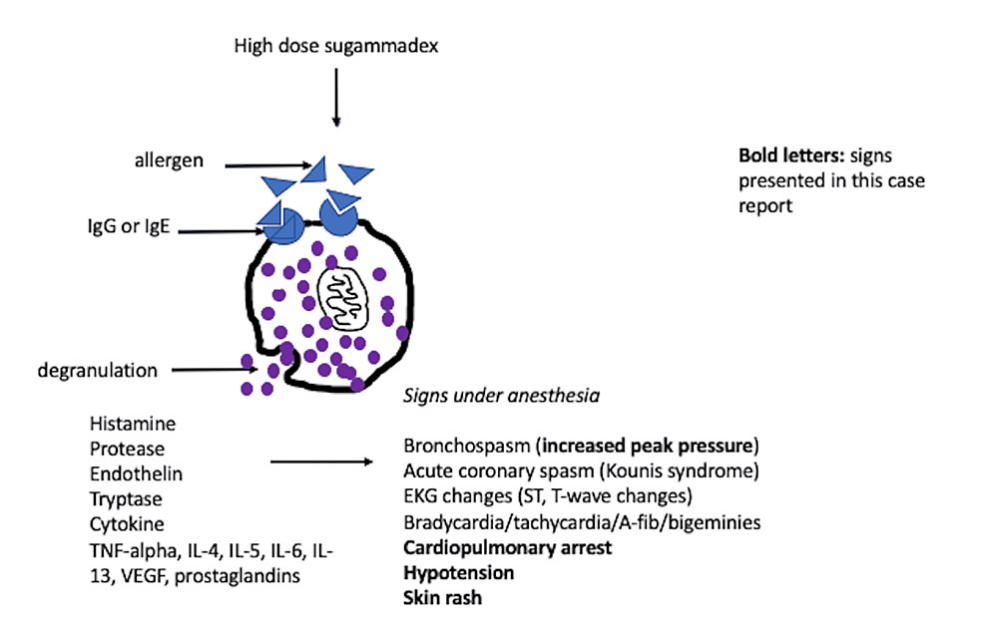
Clinical picture and mechanism of intraoperative allergic reaction due to high dose sugammadex administration VEGF - vascular endothelial growth factor

Cardiac arrest after sugammadex administration has been reported before [5-7], but the etiology is unclear. There are two possibilities: generalized vasodilation from anaphylaxis or coronary vasospasm (Kounis syndrome) [8]. The clinical picture of the case depicts a typical anaphylactic or anaphylactoid reaction because the patient developed hypotension, bronchospasm, and skin changes immediately after sugammadex administration. However, the results of her serum tryptase test sent within one hour were within normal limits. A repeat tryptase test was not done. An increase in serum tryptase indicates mast cell degranulation (Figure 1) and is predictive of IgE-mediated anaphylaxis, although tryptase can be elevated in non-IgE-mediated reactions [9]. In the literature, serum tryptase has been shown to be both elevated [10] and not elevated [11] in suspected cases of sugammadex-induced anaphylaxis. Jönsson and colleagues studied samples from people who experienced anaphylaxis after neuromuscular blocker administration [12]. They found that drug-specific IgG complexes triggered neutrophils ex vivo. Moreover, patients who had drug-induced anaphylaxis had increased circulating activated neutrophils. It is unknown if such an alternative pathway is also involved in sugammadex-induced anaphylaxis.

Our patient may have received sugammadex previously and developed an erythematous rash in the recovery unit. This information was not available to the anesthesiologist prior to the surgery. It is possible that the patient developed a profound reaction due to repeated exposure to the drug. However, the United States Food and Drug Administration reports that repeated exposure to sugammadex does not increase the risk of anaphylaxis [13]. Additionally, the patient received a relatively high dose of sugammadex (4 mg/kg). It has been hypothesized that the risk of anaphylactic reactions increases with higher doses of the drug [14]. In a double-blind, placebo-controlled study, 448 healthy volunteers were randomized to intravenous administrations of placebo, sugammadex 4 mg/kg, or 16 mg/kg and developed dose-dependent hypersensitivity (0.0%, 0.7%, vs. 4.7%) [14]. 

## Conclusions

Perioperative anaphylaxis is rare. A low number of complications makes it difficult to estimate the true incidence in a prospective study. Moreover, when it happens, anesthesiologists find it difficult to diagnose the condition due to its variable presentation or coexisting hypotension from anesthetics. It is, therefore, imperative that all suspected cases of anaphylaxis are reported so that anesthesiologists can develop strategies to prevent and improve perioperative care for this rare complication.
